# Quantitative proteome profile of water deficit stress responses in eastern cottonwood (*Populus deltoides*) leaves

**DOI:** 10.1371/journal.pone.0190019

**Published:** 2018-02-15

**Authors:** Paul E. Abraham, Benjamin J. Garcia, Lee E. Gunter, Sara S. Jawdy, Nancy Engle, Xiaohan Yang, Daniel A. Jacobson, Robert L. Hettich, Gerald A. Tuskan, Timothy J. Tschaplinski

**Affiliations:** 1 Chemical Sciences Division, Oak Ridge National Laboratory, Oak Ridge, Tennessee, United States of America; 2 Biosciences Division, Oak Ridge National Laboratory, Oak Ridge, Tennessee, United States of America; Institute of Genetics and Developmental Biology Chinese Academy of Sciences, CHINA

## Abstract

Drought stress is a recurring feature of world climate and the single most important factor influencing agricultural yield worldwide. Plants display highly variable, species-specific responses to drought and these responses are multifaceted, requiring physiological and morphological changes influenced by genetic and molecular mechanisms. Moreover, the reproducibility of water deficit studies is very cumbersome, which significantly impedes research on drought tolerance, because how a plant responds is highly influenced by the timing, duration, and intensity of the water deficit. Despite progress in the identification of drought-related mechanisms in many plants, the molecular basis of drought resistance remains to be fully understood in trees, particularly in poplar species because their wide geographic distribution results in varying tolerances to drought. Herein, we aimed to better understand this complex phenomenon in eastern cottonwood (*Populus deltoides*) by performing a detailed contrast of the proteome changes between two different water deficit experiments to identify functional intersections and divergences in proteome responses. We investigated plants subjected to cyclic water deficit and compared these responses to plants subjected to prolonged acute water deficit. In total, we identified 108,012 peptide sequences across both experiments that provided insight into the quantitative state of 22,737 *Populus* gene models and 8,199 functional protein groups in response to drought. Together, these datasets provide the most comprehensive insight into proteome drought responses in poplar to date and a direct proteome comparison between short period dehydration shock and cyclic, post-drought re-watering. Overall, this investigation provides novel insights into drought avoidance mechanisms that are distinct from progressive drought stress. Additionally, we identified proteins that have been associated as drought-relevant in previous studies. Importantly, we highlight the *RD26* transcription factor as a gene regulated at both the transcript and protein level, regardless of species and drought condition, and, thus, represents a key, universal drought marker for *Populus* species.

## Introduction

Plants frequently experience unfavorable environmental conditions that negatively influence biomass production. Periodic water deficiency is one of the most adverse environmental factors affecting plant growth and is responsible for approximately 70% of the potential agricultural yield loss worldwide [1]. Plants experience different severities of water deficit that vary due to temperature and seasonal precipitation. Importantly, plant perception of stress and the intrinsic response depends on species as well as the severity and duration of the water deficit [2]. The duration and frequency of water availability due to precipitation translates to soil moisture deficit that initiates stomatal closure and limits gas exchange in leaves. If water deficit is extensive, prolonged desiccation will lead to gross disruption of whole-plant metabolism, eventual leaf senescence, and even plant death.

It is becoming apparent that plants respond to drought stress by reprogramming cell, tissue, organ, and whole-plant metabolism to attain new developmental and physiological equilibrium to cope with water deficit [3]. At the cellular level, plants respond to drought by modifying the complex microcosm of genes, proteins, and metabolites. These exceedingly complex modifications meet the dynamic needs of the cell, allowing plants to escape, tolerate, and recover from drought stress. With the advent of ‘omics’-related technologies, large-scale investigations are beginning to reveal key biochemical and molecular mechanisms occurring during stress [4–6]. Not only do these studies validate drought-related biomarkers under varying experimental designs and species, but they also facilitate the discovery of less obvious gene, protein, and metabolite networks that respond to stress, ultimately paving the way to targeted manipulation of drought-responsive genes in plants.

The cellular proteome is under continuous modification to meet the fluctuating needs of the cell and we are just beginning to realize how differential gene expression [7] and protein flux [8] in individual cell types translate into the organization and dynamics of the proteome. Nonetheless, knowledge of the abundance and stoichiometry of proteins provides considerable insight into the functional state of a cell, and, therefore, necessitates the need for quantitative proteomics. The proteomes of several model plants have been intensively investigated and provide considerable information into which proteins respond to water deficit [9]. As a model plant that brings insights into phenomena specific to trees, *Populus* plants are known to be among the most sensitive woody plants to water [10]. Several studies on *Populus* species have been conducted to characterize gene regulation underpinning the complex responses to drought [11, 12]. While several gel-based studies have provided relevant insight into the proteomes of *Populus* species under drought stress, the realistic scale of these analyses has been limited to a few hundred proteins [13–16]. Therefore, a large-scale, systematic analysis that quantitatively describes the proteome of a *Populus* species under drought stress would provide a critical resource to the genetic improvement of *Populus*, which in turn would improve its sustainability as a biofeedstock for biofuels and bioproducts.

In general, Poplars (*Populus sp*.) are highly productive tree species that have been identified as potential woody biomass feedstocks for bioenergy production [17]. Eastern cottonwood (*P*. *deltoides*) has a widespread distribution in the USA that includes land east of the Mississippi River, particularly in the Southeast, the North Central States, where it is has been cultured in plantations both as a pure species and as interspecific hybrids with *P*. *nigra*, and in the Pacific Northwest, where it has also been cultured as interspecific hybrids with *P*. *nigra* and *P*. *trichocarpa*, as well. Among these poplar species, *P*. *deltoides* is the most drought tolerant, exhibiting both low osmotic potential and the capacity for osmotic adjustment (lowering of osmotic potential) in response to drought and it contributes these traits to its hybrid progeny [18]. Given such attributes and that drought stress is the greatest limitation to *Populus sp*. productivity, *P*. *deltoides*, is an ideal North American poplar species to determine the underlying bases of its drought tolerance capability.

Herein, we sought to generate comprehensive proteomic profiles to better capture the dynamic regulation of protein abundance in *Populus deltoides* leaves under water deficit conditions. More importantly, most previous proteomic studies only exposed *Populus* plants to short periods of dehydration shock. The sudden onset of severe drought is unlikely to reflect what naturally happens to soil-grown plants. Therefore, rather than exposing plants to severe dehydration via the application of concentrated osmotica (e.g., polyethylene glycol), we chose to monitor plants exposed to prolonged dehydration. For the progressive drought experiment, herein referred to as acute drought, water was withheld from the plants until symptoms of wilting were observed. From this drought treatment, proteome changes that occur because of declining leaf water potentials, which consequently leads to worsening plant health, were used to provide new insights to biological pathways that respond to severe drought conditions. Because periods of drought stress, followed by post-drought re-watering periods are highly important in tree survival, we also measured proteome changes across a cyclic drought treatment to better understand drought recovery and coping mechanisms. As first noted in a meta-analysis of microarray experiments comparing different water deficit-related treatments [4], one consequence of analyzing multiple treatment conditions is that we expect only a few differentially abundant proteins to be common to both treatments. Therefore, a primary objective of this study was to identify an overall integrative picture of the conserved drought markers, but also meaningful divergences in functional behavioral responses between the two drought experiments. To this end, we identified the protein abundances that are regulated in response to both drought conditions and highlight the proteome changes that overlap with previously published proteomic and transcriptomic results. Moreover, we describe distinguishing proteome changes between the two drought experiments presented herein, with a particular focus on those associated with stomatal behavior.

## Materials and methods

### Plant materials and culture conditions

Eastern cottonwood poplar (*Populus deltoides* ‘WV94’) plantlets in leach tubes were obtained from ArborGen (Summerville, SC). After 12 weeks, plants were transferred to 1-gal containers at Oak Ridge National Laboratory (ORNL) using ArborGen LLC’s standard hardwood mix: peat:vermiculite:perlite (2:2:1 v:v:v) supplemented with the following fertilizers (milliliters/gallon): Osmocote (12), Bone Meal (4), Gypsum (4), and Dolomite (2), and placed in ORNL greenhouses (19°C, no supplemental lighting). Plants received ambient lighting through the Exolite wall panels of the solar greenhouse. Plants were fertilized with Peter’s 20-20-20 NPK fertilizer at 300 ppm per month. Plants were then regenerated from cuttings from this stock as needed for subsequent drought experiments as described in the following sections. Importantly, the stress levels implemented below were chosen based on extensive analysis of conducting controlled drought stress trials on poplar in both greenhouse and field conditions. Field capacity is widely accepted to be at ~-0.1 MPa, and this is what we have routinely observed in both field drought studies [18] and greenhouse drought studies, including this study. Stress initiation in well-watered poplars ranges from -0.3 MPa [18] to -0.5 MPa in poplar [19] and other fast-growing hardwood species [20]; hence we selected -0.5 MPa for stress initiation with the severe stress threshold at -0.8 MPa. The severe stress was shifted lower with drought exposure due to acclimation to -1.0 MPa as has been observed for poplar hybrids in the field [21].

### Acute drought experiment

Nine (plants 1–9) six-month-old *Populus deltoides* ‘WV94’ plants were clonally propagated from 15-cm long cuttings in 3.8 L pots for six months. Plants were well watered (kept near field capacity; ~-0.10 MPa) until the start of the acute drought experiment, when the watering ceased and all plants were allowed to dry down without watering for 8 consecutive days. The plants at the start of the experiment had an average height of 87.8 cm and diameter of 0.31 cm, resulting in a stem volume of 8.8 cm^3^. Leaves (leaf plastochron index (LPI) 9–12) of clonal replicates 1–3 were sampled for predawn leaf water potential. Plants 4–6 were similarly sampled on days 1 (BD), 4, and 7 (B7). Plants 7–9 were similarly sampled on days 2, 5 (B5), and 8 (B8). Thus, at each time point three biological replicates were sampled. Predawn leaf water potential was measured with a Scholander pressure cylinder.

### Cyclic drought experiment

Twenty six-month-old *P*. *deltoides* ‘WV94’plants were clonally propagated from 15-cm long cuttings in 3.8 l pots. Similar to the plants in the acute drought study described above, plants in the cyclic drought study were of similar stature and watered daily before the implementation of the drought treatments. Eight plants continued to be watered daily to keep them at field capacity throughout the duration of the study and were considered the well-watered control plants with water potentials ≥-0.10 (±0.01) MPa. Twelve additional plants were selected for the cyclic drought treatments. The target predawn leaf water potentials before rewatering were -0.50 MPa and -0.80 MPa for the mild and severe drought stress treatments, respectively. Similar to the acute drought experiment, predawn leaf water potential was measured with a Scholander pressure cylinder between 4:30–6:00 on three trees per treatment using mature leaves. Plants in drought treatments were re-watered once they achieved the target predawn water potentials, which occurred on days 6, 12, and 20 after the initiation of the cyclic drought experiment. Plants in the severe drought treatment were the same as those in the mild stress treatment, but they were allowed to continue to dry down for an additional day after the second and fourth drought cycles to achieve the target leaf water potentials before being sampled and then removed from the study without further treatment or sample collection. The cyclic drought treatments were conducted for a 30-day period with the last predawn water potential for the cyclic severe drought occurring early on day 31. Leaves sampled for predawn leaf water potentials were LPI 9–12 (fully expanded, mature leaves). Leaf LPI 10 was collected midday for ‘omics measurements at the end of drought cycle 2 (day 6 for mild drought and control plants, day 7 for severe drought) and cycle 4 (day 9 for mild drought and control plants, day 10 for severe drought) and then processed, as described below. There were 4 biological replicates for the drought and well-watered treatments at each sampling time point.

### Protein extraction and digestion

Cell lysis, protein extraction and digestion were performed as previously described [22], albeit with minor modifications. Leaf tissues were ground under liquid nitrogen using a mortar and pestle. A 2–3 g sample of ground tissue was suspended in boiling SDS lysis buffer (4% SDS in 100 mM of Tris-HCl), boiled for 5 min, sonically disrupted (30% amplitude, 10 s pulse with 10 s rest, 2 min total pulse time) and boiled for an additional 5 min. Crude protein extract was pre-cleared via centrifugation, and quantified by BCA assay (Pierce Biotechnology). One milligram of crude protein extract was then precipitated by trichloroacetic acid, pelleted by centrifugation and washed with ice-cold acetone to remove excess SDS, as previously described. Pelleted proteins were resuspended in 250 μL of 8 M urea, 100 mM Tris-HCl, pH 8.0 using sonic disruption to fully solubilize the protein pellet and incubated at room temperature for 30 min. Denatured proteins were reduced with DTT (10 mM) and cysteines were blocked with iodoacetamide (20 mM) to prevent reformation of disulfide bonds. Samples were digested via addition of two aliquots of sequencing-grade trypsin (Promega, 1:75 [w:w]) at two different sample dilutions, 4 M urea (overnight) and subsequent 2 M urea (5 h). Following digestion, samples were adjusted to 200 mM NaCl, 0.1% formic acid and filtered through a 10 kDa cutoff spin column filter (Vivaspin 2, GE Health) to remove under-digested proteins. The peptide-enriched flow through was then quantified by BCA assay, aliquoted and stored at −80°C.

### LC-MS/MS

For the analysis of the proteome samples, 50 μg of each peptide mixture were bomb-loaded onto a biphasic back column packed with ~5 cm strong cation exchange (SCX) resin followed by ~3 cm C18 reversed phase (RP) (Luna and Aqua respectively, Phenomenex). Each peptide-loaded column was first washed off-line to remove residual urea and NaCl and then placed in-line with an in-house pulled nano-electrospray emitter (100 micron ID) packed with 15 cm of C18 RP material and analyzed via 24-hr MudPIT 2D-LC-MS/MS as previously described [22]. Peptide sequencing analysis was performed with an LTQ-Orbitrap-Velos-Pro mass spectrometer (ThermoFischer Scientific). Data acquisition was managed by XCalibur version 2.1. Mass spectra were acquired in a data-dependent “top 20” mode: each survey scan was followed by MS/MS spectra of the twenty most abundant precursor ions (3 m/z isolation window). High-resolution (30,000 at m/z 400) MS1 spectra were acquired in the Orbitrap analyzer. For peptide fragmentation, monoisotopic precursor selection and charge state rejection of singly-charged precursors were enforced and normalized collision energy of 35% was used for CID. Each fragmented precursor ion was dynamically excluded from further targeting for 60 seconds. A dynamic exclusion repeat of 1 and a mass width of 20 ppm were applied to maximize peptide sequencing.

### Peptide identification and protein inference

All experimental MS/MS spectra were compared to theoretical tryptic peptide sequences generated from a FASTA database containing the full protein complement of the *Populus trichocarpa v3*.*0* database (https://phytozome.jgi.doe.gov), appended with chloroplast, mitochondria and common contaminant proteins [23]. A decoy database, consisting of the reversed sequences of the target database, was appended in order to discern the false-discovery rate (FDR) at the spectral level. For standard database searching, the peptide fragmentation spectra (MS/MS) were searched with MyriMatch algorithm v2.2 [24]. MyriMatch was configured to derive fully-tryptic peptides with the following parameters: unlimited missed cleavages, parent mass tolerance of 10 ppm, a fragment mass tolerance of 0.5 m/z units, a static modification on cysteine (+57.0214 Da), an N-terminal dynamic modification of (+43.0058 Da), and a dynamic modification corresponding to an oxidation (+15.9949 Da) of methionine. For protein inference and post-search filtering, database search results for each sample were merged together in IDPicker v3.1.643 [25]. To achieve an experimental false discovery rate (FDR) of <1% at the peptide level, we enforced protein identifications to have at least two distinct peptide identifications and a minimum of 12 spectral count across the all runs. To deal with the redundancy associated with the *Populus* protein database, all proteins in the FASTA database were grouped by sequence similarity (≥90%) using the UCLUST component of the USEARCH v5.0 software platform [26]. As described previously [27], grouping proteins by this conservative level of sequence identity serves to 1) maintain biologically-relevant peptide information that would have otherwise been lost due to proteomic redundancy and 2) eliminate most ambiguity inherent to higher eukaryotes (i.e., peptides mapping to multiple protein isoforms resulting from whole-genome duplication events). The protein data has been deposited at ProteomeXchange [28] as a ‘complete’ submission.

### Label-free quantification

Peptide abundance extracted ion chromatogram intensities were extracted from MS data using IDPQuantify [29]. Log2 transformation of peptide intensities was performed to rescale the data sets. To extract ion intensities from each chromatogram, we used the following IDPQuantify settings: low and upper retention time of 30 s; lower and upper chromatogram mass tolerance of 10 ppm; no retention time alignment. Protein abundances were calculated by summing together their respective peptide intensities. Log 2-transformed protein intensities were loaded into the InfernoRDN v1.1 [30] software and values were normalized by median centering adjustments across the global dataset.

### Statistical analysis for differential abundances

For this study, we performed pair-wise comparisons between the well-watered controls (i.e., samples “C” and “BD” for the cyclic and acute experiments, respectively) and the water deficit samples as we aim to identify the differences between varying water potentials and the control and not the overall change in protein abundances across the entire experiment. Student’s *t*-tests were utilized to identify differences in quantitative abundances between conditions using the computational program Perseus [31]. To improve the robustness of the analysis, we limited the analyses to only those proteins observed in at least two biological replicates of at least one sample for each pairwise comparison. For each pairwise comparison, proteins having null abundances values (originally zero) were imputed with random numbers from a normal distribution, whose mean and standard deviation were chosen to best simulate low abundance values below the noise level (Width = 0.3; Shift = 2.5). A protein was categorized as having a significant abundance difference between a well-watered control and water deficit condition if it passed the significance threshold requiring a p≤0.05 and absolute value of log2 fold-change difference >1.

### Gene ontology enrichment

Whole-genome gene ontology (GO) term annotation was performed using Blast2GO [32] with a blastp E-value hit filter of 1×10^−5^, an annotation cutoff value of 55 and a GO weight of 5. Using ClueGO [33], observed GO biological process were subjected to the right-sided hypergeometric enrichment test at medium network specificity selection and p-value correction was performed using the Holm-Bonferroni step-down method [34]. There were a minimum of 3 and a maximum of 8 selected GO tree levels, while each cluster was set to include a minimum of between 3% and 4% of genes associated with each term. For minimal reporting of functional groups, GO term fusion and grouping settings were selected to reduce GO term redundancy and the term enriched at the highest level of significance was used as the representative term for each functional cluster. The GO terms at adjusted p≤0.05 were considered significantly enriched.

## Results and discussion

### Identification and quantification of protein abundances responding to specific water deficit stages in mature leaves

**Fig 1:**
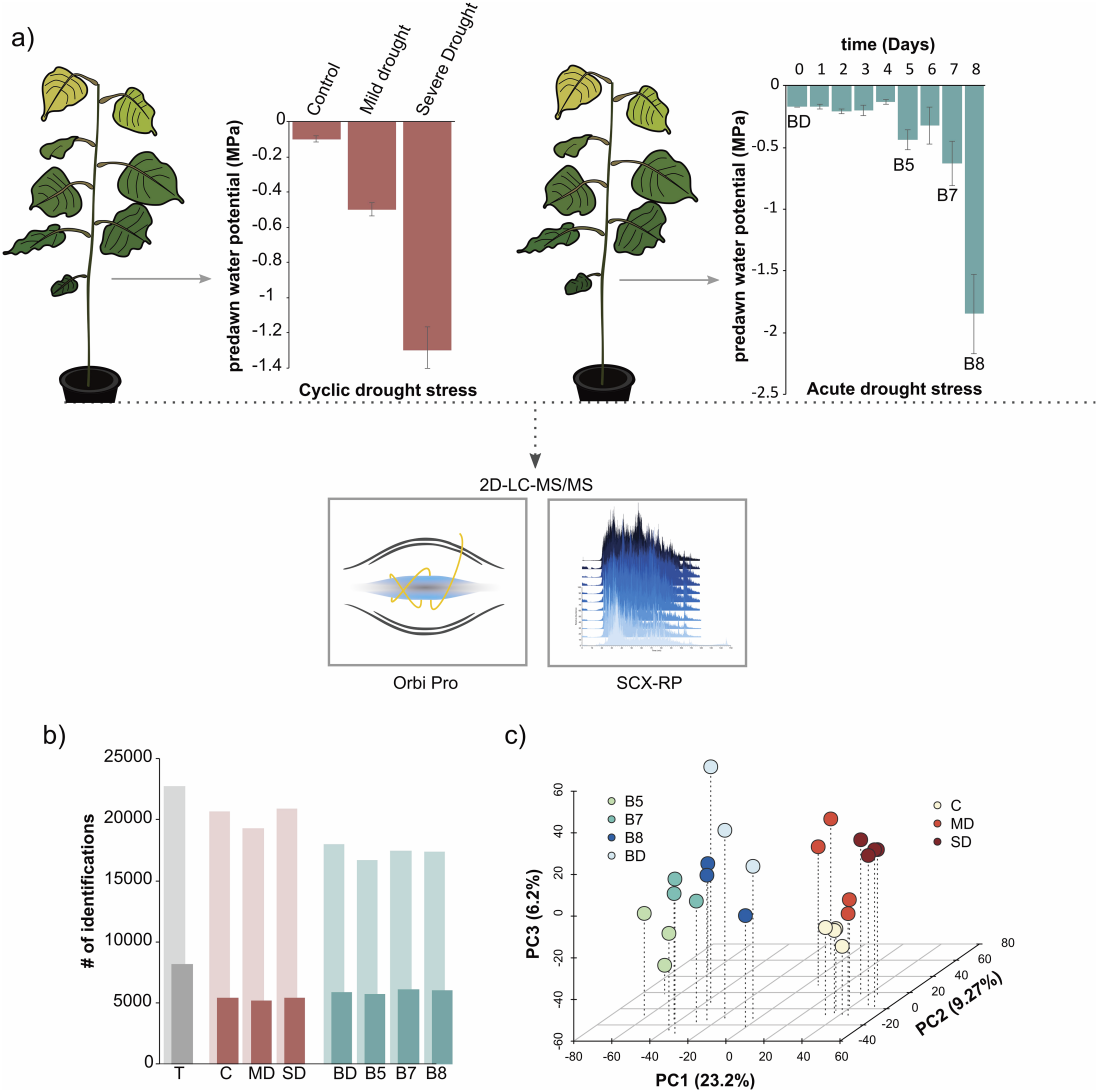
Label-free quantitative proteomic analysis of water deficit in *P*. *deltoides* leaves. **(A)** Large-scale characterization of proteome behavioral response to prolonged cyclic drought (red) vs acute drought (blue) treatment was achieved by two-dimensional liquid chromatography tandem mass spectrometry (2D-LC-MS/MS). For the cyclic drought treatment, three samples were collected to profile a well-watered leaf versus mild and severe drought deficit conditions. The acute drought stress treatment provides a complementary perspective, in which four samples capture prolonged water deficit conditions across an 8-day period. The average water potential and standard error of the mean are provided for each sample. **(B)** The total number of identified proteins and protein groups are highlighted, with the lighter color shade and the offset darker color representing the number of proteins and protein groups, respectively. **(C)** Partial least squares analysis highlights the discrete grouping of biological replicates and the two treatments.

A cyclic watering (4 cycles) experiment and a progressive slow-drying experiment were carried out in 6-month-old *P*. *deltoides* plants (Fig 1). To determine the severity of the drought stress, daily measurements of pre-dawn leaf water potentials were carried out. Leaves were sampled from a well-watered stage and water potentials representative of mild-to-severe stress for the cyclic (Fig 1a) and prolonged acute (Fig 1b) drought stress experiments. Overall, we identified 108,012 non-redundant peptide sequences across both experiments. In total, the peptides mapped to, and, thereby, provided insight into the quantitative state of 22,737 *Populus* gene models and 8,199 functional protein groups in response to varying water deficit stages (Table A in S1 File). This dataset surpasses the depth of analysis achieved by the often-used two-dimensional gel electrophoresis and provides the largest and most comprehensive drought-related proteome dataset for leaves in *Populus* to date.

In general, the two treatments greatly overlapped in the proteins identified, with 98% of all identified proteins observed in both datasets. Despite this similarity, multivariate partial least squares analyses found a clear distinction between all cyclic and prolonged proteins in the first component (Fig 1c). Based on pairwise t-tests, we observed 2,287 differentially abundant proteins (1,077 protein groups) in the cyclic experiment and 2,943 differentially abundant proteins (1,376 protein groups) in the acute experiment (Table B in S1 File). Overall, the datasets together provided a list of 4,754 proteins that responded in abundance to water deficit treatment. Interestingly, the two treatments only shared 476 overlapping differentially abundant proteins.

**Fig 2:**
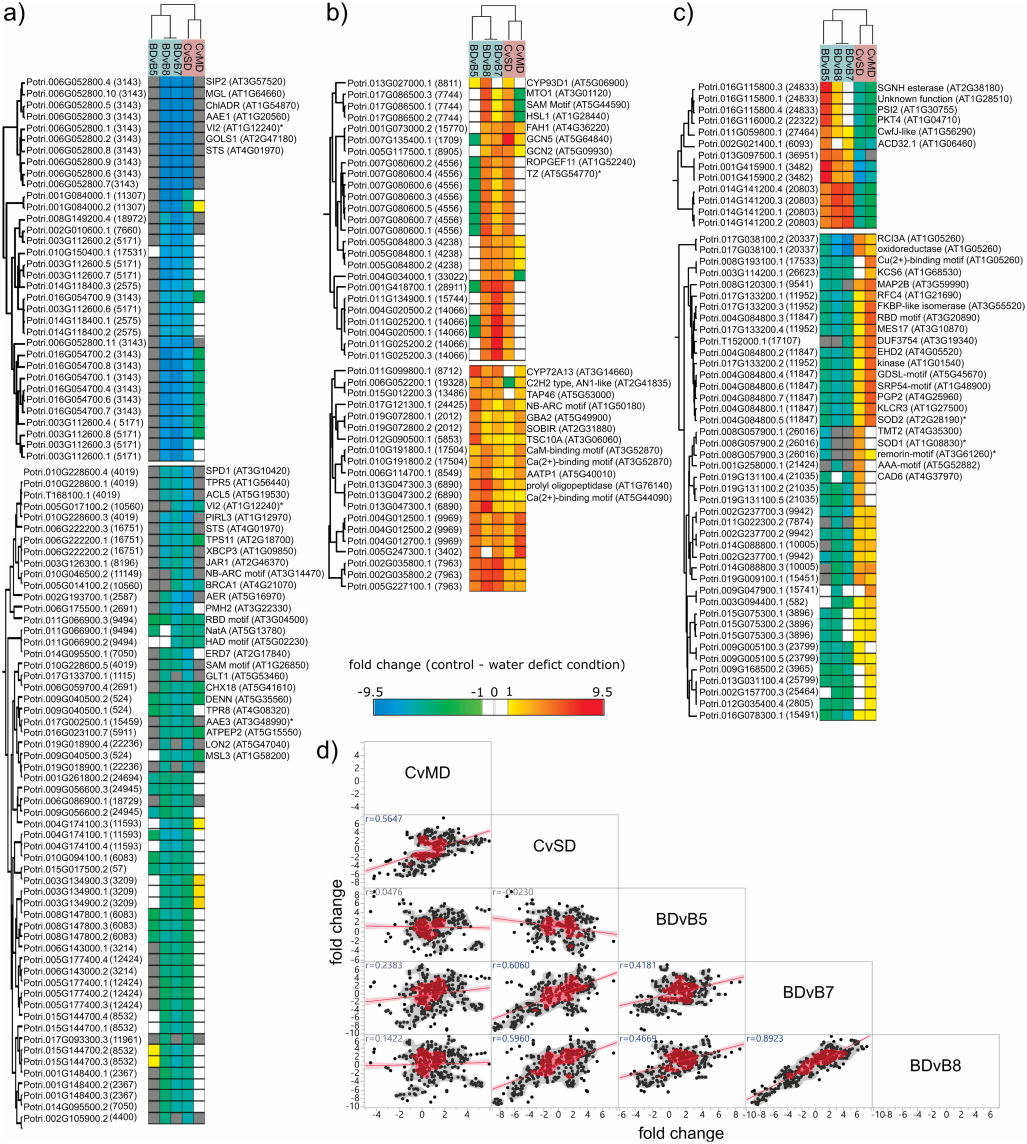
Differential protein abundance behaviors observed in both experiments. Two-way hierarchical clustering (Euclidian) of Log2 fold changes for each pairwise comparison was performed in the Perseus software for on only the differentially abundant proteins observed in both experiments. Here, we highlight discrete clusters representing those proteins that behaved similarly **(A-B)** or differently **(C)** between the two experiments. Proteins not observed in a pairwise comparison are represented by a grey color. In parentheses following each *Populus* accession number is the protein group index. To the right of each cluster, we provided the observed *A*. *thaliana* homologs. **(D)** A scatterplot matrix of protein fold changes highlights the similarity between the water deficit treatments. A fitted line, nonparametric density and Pearson correlation coefficient is provided for each scatterplot.

For these overlapping proteins, we performed hierarchical clustering (Euclidean distances) and calculated Pearson correlation coefficients for the fold changes across each pairwise comparison (Fig 2) to provide insight into how these protein abundance behaviors compared between experiments. In general, we observed several proteins that behaved similarly (Fig 2a and 2b), in which the protein was up- or down-regulated in both experiments, as well as several that had opposing behaviors (Fig 2c). Moreover, the scatterplot matrix (Fig 2d) shows that day 7 and day 8 of the prolonged drought experiment elicited similar behavior responses, and these were most like the severe water deficit condition in the cyclic experiment.

To relate these findings to past and future studies, we investigated which proteins have been described in a recent review [9] that comprehensively detailed drought-responsive proteins across 44 studies. The proteins with *A*. *thaliana* orthologs and gene aliases have been marked and listed on the right-hand side of Fig 2a and 2c. Interestingly, only a few of these proteins were reported in the recent review article, suggesting that the current dataset provides new insights into water deficit-relevant proteins. We suspect this can be mostly explained by the significant measurement depth of this drought-related study.

Nonetheless, among the protein abundances that were up-regulated in response to drought in both experiments, we identified proteins related to peroxisome-related processes (e.g., *ChlADR*, *AAE1*, *AAE3*, *LON2*), stomatal behavior (*SIP2*, *VI2*), oxidative damage and signaling (e.g., *GOS1*, *STS*, *PIRL3*, *TPS111*, *JAR1*, *NB-ARC*, *AER*, *NAA10*, *MSL3*), cell wall modifications (e.g., *ACL5*), and gene regulation (e.g., *BRCA1*, *ATPEP2*). The protein abundances that were down-regulated in response to both drought experiments were primarily associated with oxidation-reduction processes (e.g., *CYP93D1*, *CYP72A13*, *MTO1*), growth and development (e.g., *HSL1*, *GCN2*, *GCN5*, *ROPGEF11*, *TAP46*) and acyl-lipid metabolism (e.g., *FAH1*, *TSC10A*). It is important to note the regulated proteins that support previous studies that identified a gene as drought-responsive (e.g., *LON2* [35] and *ERD7)* [36, 37].

These findings present additional insights into chemical surveillance mechanisms in response to drought. For example, a recent study [38] observed that Nα-terminal acetylation, which is a general cellular surveillance mechanism in higher plants that contributes significantly to the response to abiotic stresses, decreases significantly after drought stress and that the *NatA* complex is coincidently down-regulated by the phytohormone abscisic acid. Interestingly, these *Populus* protein abundances, which have ~83% sequence identity to the *A*. *thaliana* homolog, were significantly up-regulated in both the cyclic (C vs. SD, fold change = 7.3X) and acute drought (BD vs. B5, fold change = 18.4X) experiments. As such, these results imply that there is much more to learn about this important switch that controls aspects of metabolism, development, and cellular stress responses downstream of abscisic acid, and that the Nα-terminal acetylation cellular profile may differ in the circumstances presented herein.

While the above subset of proteins provides new insight into core drought-responsive mechanisms in a *Populus* species, the proteins that have opposing behaviors, on the other hand, reveal key differences in how *Populus* leaves functionally respond to either a cyclic or prolonged acute drought stress. Among the protein abundances that are up-regulated in the cyclic experiment and down-regulated in the acute drought experiment, we observed several functionally related to acyl-lipid metabolism (e.g., *SGNH hydrolase-type esterase*, *PKT4*) and growth and development (e.g., *PSI2*, *ACD32*.*1*). Importantly, we observed a protein of unknown function (AT1G28510) that has been reported to be involved in several drought studies [39, 40] and should be a target of future investigations to better understand this protein’s functional role. Within the subset of protein abundances that are down-regulated in the cyclic experiment and up-regulated in the acute drought experiment, we observed several functionally related to oxidation-reduction processes (e.g., *RCI3A*), cell wall modifications (e.g., *KCS6*), protein processing (e.g., *MAP2B*), and growth and development (e.g., *RFC4*, *MAS17*, *EHD2*). Interestingly, several of the down-regulated protein abundances have *Arabidopsis* homologs (AT1G68530, AT1G08830, AT5G52882, AT4G37970) with implications for cellular dehydration stress memory [41], suggesting these *Populus* proteins may belong to an adaptive response mechanism.

In addition to mining already available drought-relevant proteomic studies in general, we investigated whether any of these proteins have been highlighted as potential candidate drought-responsive genes in a recently published transcriptome study of *Populus* species [12]. Of the protein abundances observed to be significantly regulated in both experimental conditions presented herein, a substantial number of proteins (939) were also reported as having significant gene expression responses to prolonged drought and were associated with drought escape, avoidance and tolerance strategies in another *Populus* species, black poplar (*Populus nigra*). Importantly, these identified proteome regulation events that overlap with this recently published transcriptome study reveal key genes that have observed regulation at the transcript level (Yildirim et al. 2017) and protein level and this regulation occurs irrespective of species (Table C in S1 File). For example, one of the most up-regulated proteins in both prolonged acute drought and cyclic drought datasets was also one of the most up-regulated transcripts in all three genotypes characterized by their tolerance to prolonged drought, sensitive (S), moderate resistance (MR), and resistant (R). As such, this specific gene is highly regulated at both the transcript and protein level regardless of the not only *Populus* species but also the drought condition. This gene (*RD26*; Potri.011G123300) was recently characterized as a transcription factor that is known to be induced in response to desiccation [42]. Leaf abscission, in general, is known to be a primary mechanism for drought avoidance in *Populus* species to pervert water loss via transpiration [43]. Therefore, this transcription factor represents a major and universal response to drought for *Populus* species and should be investigated further.

### Association of differential protein abundance behavior with functional terms

**Fig 3:**
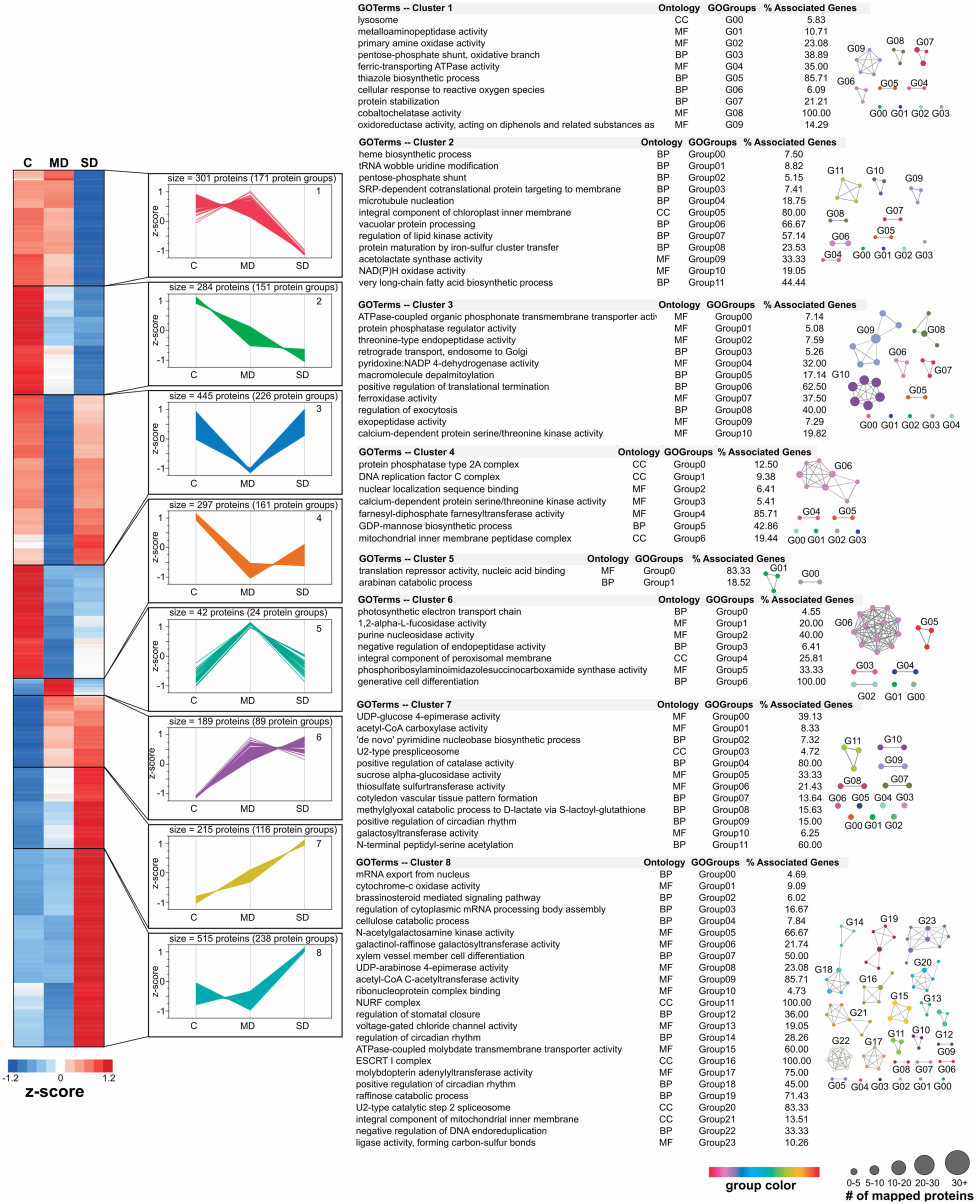
Functional profiles for differential abundance patterns observed in cyclic drought treatment. To identify different behavioral responses in protein abundance across the cyclic drought experiment, hierarchical clustering (Fast Ward method) was performed on only the differentially abundant proteins. The Figure of Merit (FOM) algorithm was used to estimate an appropriate number of clusters, resulting in 8 protein cluster groups. To capture general patterns without considering absolute abundance levels, standardized z-scores [(abundance—mean)/ standard deviation] were used for to illustrate the heat map and line plots. To the right of each clusters line plot, we highlight their minimized, non-redundant list of significantly enriched GO terms identified by the ClueGO software. For each representative GO term, we provide the ontology category, the GO functional group index, as identified by ClueGO, and the percentage of proteins observed out of the total associated for each GO term in the *Populus* proteome. The significantly enriched GO terms for each cluster were also illustrated as a network model, in which the size of each GO term represents the number of associated proteins.

**Fig 4:**
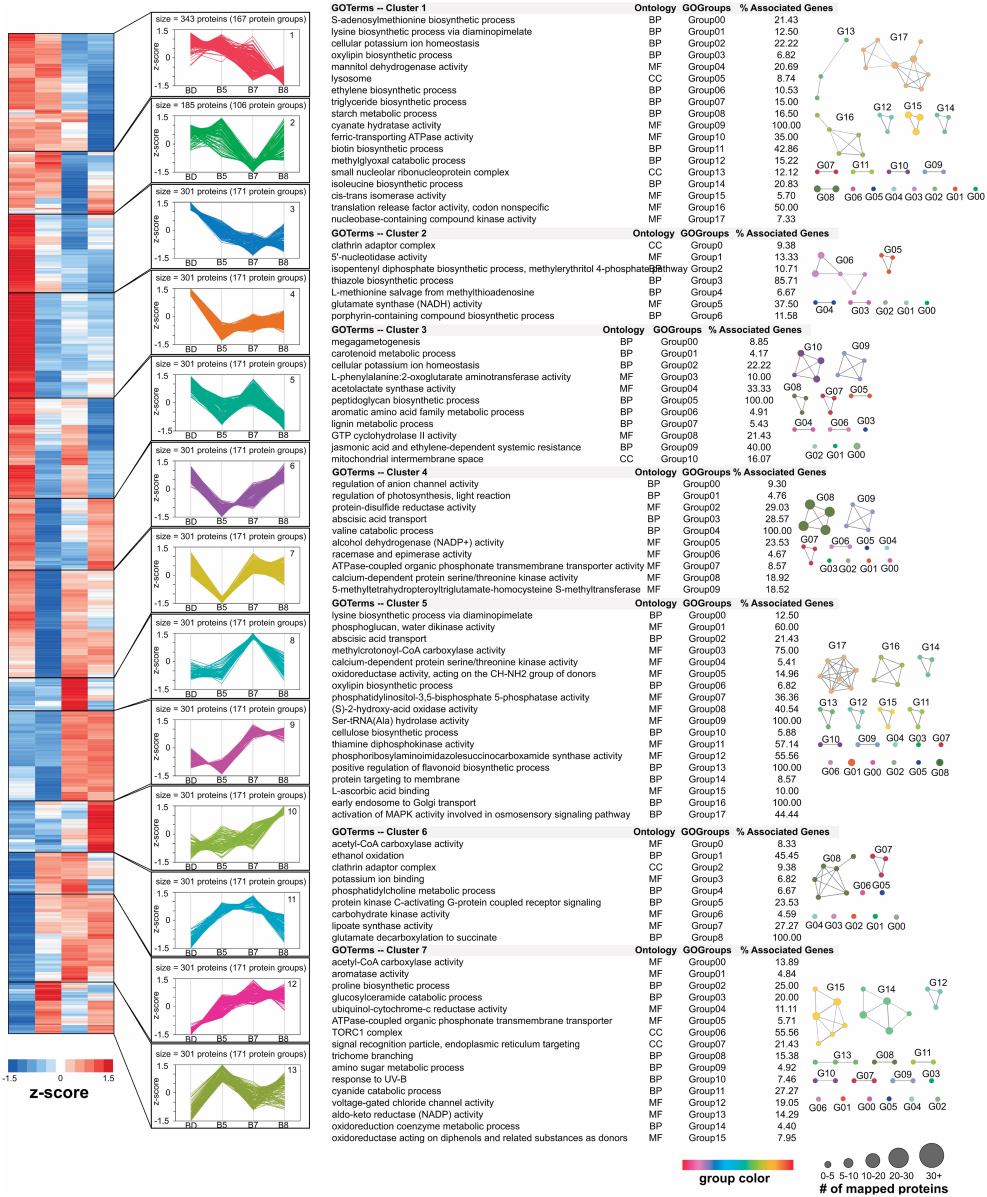
Functional profiles for differential abundance patterns observed in acute drought treatment. To identify different behavioral responses in protein abundance across the acute drought experiment, hierarchical clustering (Fast Ward method) was performed on only the differentially abundant proteins. The Figure of Merit (FOM) algorithm was used to estimate an appropriate number of clusters, resulting in 13 protein cluster groups. To capture general patterns without considering absolute abundance levels, standardized z-scores [(abundance—mean)/ standard deviation] were used to illustrate the heat map and line plots. To the right of each clusters line plot, we highlight their minimized, non-redundant list of significantly enriched GO terms identified by the ClueGO software. For each representative GO term, we provide the ontology category, the GO functional group index, as identified by ClueGO, and the percentage of proteins observed out of the total associated for each GO term in the *Populus* proteome. The significantly enriched GO terms for each cluster were also illustrated as a network model, in which the size of each GO term represents the number of associated proteins.

**Fig 5:**
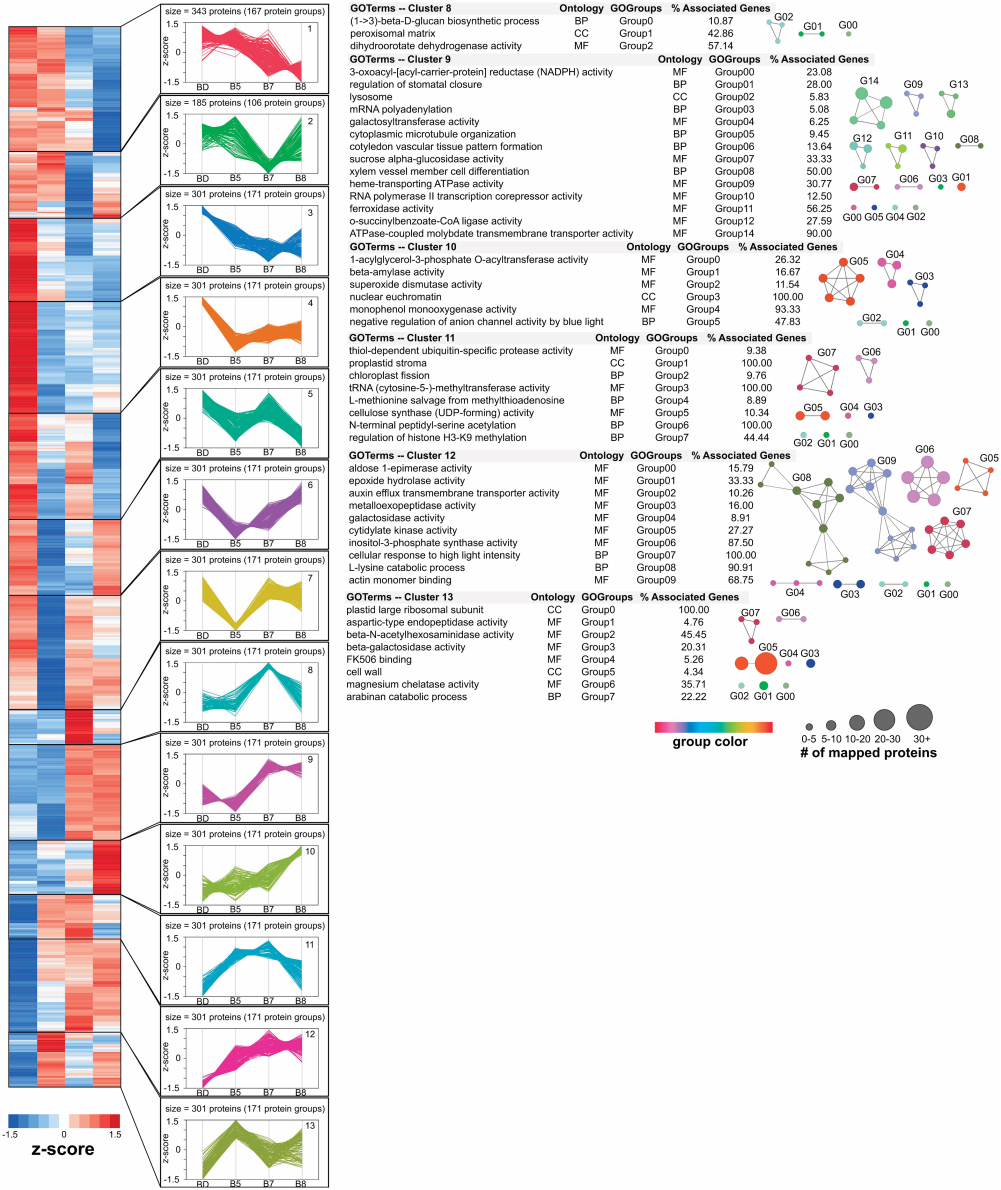
Functional profiles for differential abundance patterns observed in acute drought treatment (continued).

To provide a proteome-wide perspective on the distinct functions temporally executed by the cells to coordinate biochemical, developmental, and physiological changes in response to varying water deficit conditions, we used hierarchical clustering (Fast Ward method) to capture the orchestrated responses of the differential cyclic (Fig 3; Table C in S1 File) and prolonged acute drought stress proteomes (Figs 4 and 5; Table D in S1 File). This bioinformatics analysis only considered those differential protein abundances with an observed *p*≤0.05 and an absolute value of Log2 fold change>1. For each discrete cluster identified, we used ClueGO [33] as a bioinformatic tool to analyze the GO terms for over-representation to capture functional changes across the various water deficit conditions. Overall, we identified 8 and 13 clusters for the cyclic and acute drought stress experiments, respectively. In general, these clusters provide insight into the different patterns of protein abundances in response to water deficit over time. To associate each cluster with over-represented biological processes, molecular functions, or cellular compartments, we tested for over-representation of GO terms within each cluster using the ClueGO plugin. Across the 8 clusters in the cyclic experiment, we identified a total of 629 over-represented GO terms, including 106 for cellular compartment terms, 188 molecular function terms, and 335 for biological process terms (Table E in S1 File). For the 13 clusters in the acute experiment, we identified a total of 920 GO terms, including 71 cellular compartment terms, 330 molecular function terms, and 519 biological process terms (Table F in S1 File). To better explore the complexity of the proteome changes induced by the different water deficit conditions, we reduced functional redundancy within the list of over-represented GO terms using GO term fusion, an advanced setting in ClueGO that identifies terms in parent-child relationship that share similar genes and retains the most representative parent or child GO term. The representative GO terms (adjusted p≤0.05) for biological processes, cellular components, and molecular function categories were visualized using the Cytoscape suite and the associated functional groups are highlighted adjacent to each cluster in Figs 3–5.

### Functional overview of acute and cyclic drought experiments

A plant’s reaction to drought stress involves complex mechanisms that are species-specific and fine-tuned by the intensity and duration of a drought. In brief, after an initial decrease in water potential, proteins associated with sensing and signaling trigger gene regulation mechanisms that initiate a concerted cascade of response mechanisms, leading to alterations in metabolism, antioxidant processes, membrane modeling, and stomatal behavior. As a plant adapts to tolerate the imposed stress, the plants’ metabolic performance begins to decline, which is a result of stomatal limitation: the major factor for photosynthetic reduction and inhibited plant growth [44]. As such, a robust assessment of the functional changes associated with stomatal movement under water deficit is needed in *Populus*. From the molecular point of view, how prolonged drought and cyclic drought differ during changes in stomatal behavior is relatively unknown. Therefore, the scope of this analysis was limited to the characterization of the functional terms that co-occur with changes in stomatal behavior. For both experimental datasets, the GO biological process term, “regulation of stomatal closure”, was expectedly found to be over-represented in a cluster showing up-regulated protein abundances in response to severe drought.

Within the cyclic drought proteome dataset, this GO term was observed in cluster 8, which shows a significant increase in protein abundance in the SD sample, which had an average water potential of -1.26 MPa. Within this cluster, we observed 84 over-represented fusion GO terms, and, after applying a kappa score to define functional groups between GO terms, we identified 24 representative GO terms; these terms belong to 515 proteins (238 protein groups). Among the proteins were a diverse set of protein kinases (26 proteins; 13 protein groups), including a kinase (Potri.012G097000, *SNF1-related kinase 1;* up-regulated ~47 fold) that is activated in response to energy-depleting stress conditions to regulate an energy-saving program that maintains energy homeostasis while also gatekeeping important developmental transitions for optimal growth and survival [45]. A large set of transcription factors (40 proteins; 13 protein groups) were also identified in cluster 8. The top two most up-regulated transcription factor abundances were a homolog of a NAC transcription factor (Potri.012G097000.1, *RD26;* up-regulated ~100 fold) that is induced in response to desiccation and a basic-leucine zipper transcription factor (Potri.009G125400, *BZIP29*; up-regulated ~32 fold) that has been implicated in osmosensory responses [46] and identified in guard cells [47]. Together, the list of kinases and transcription factors provide new insights into the molecular regulators that are directly or indirectly associated with stomatal behavior in *Populus* during cyclic drought stress. Moreover, the cyclic drought experiment provides a valuable perspective on proteome changes influenced by drought effects of previous cycles; changes likely reflect a strategy to cope with a fluctuation in water status. Most associated proteins correlating with stomatal regulation function in phytochrome-mediated photomorphogenesis [48] (e.g., Potri.016G018300, *FHY3*; up-regulated ~31 fold) and circadian signaling [49] (e.g., Potri.004G168400, *XCT*; up-regulated ~11 fold) that impact developmental and physiological processes, including chloroplast osmolarity [50] (e.g., Potri.002G105900, *MSL2*; up-regulated ~29 fold) and chloroplast development [51] (e.g., Potri.015G091700, *TAC10*; up-regulated ~16 fold). In addition, we identified a protein (Potri.011G112400, *STO1*; up-regulated ~15 fold) that is localized to the chloroplast stroma and thylakoid membrane, and recently implicated in stress responses that correlated with changes in morpho-physiological traits [52]. Interestingly, many of the above proteins have functions, for example, chlorophyll biosynthesis and recovery of chloroplast structure, that are very important for seedling photomorphogenesis [53]. It is widely acknowledged that seedling germination development and growth are potentially the most critical stages for adaptive water stress coping mechanisms. As such, the observed proteins in this dataset strongly suggest the identification of several key adaptive strategies to combat drought stress in *Populus*.

Within the prolonged acute drought proteome dataset, the GO biological process term, “regulation of stomatal closure”, was observed in cluster 9 and shows a significant increase in protein abundance occurring at day 7 (B7) of water deficit, which had an average predawn leaf water potential of -0.63 MPa. Notably, this water potential is substantially higher than the cyclic experiment, suggesting that plants that have already experienced recent drought treatments exhibit a delayed molecular response in the regulation of stomatal behavior relative to the prolonged acute drought treatment. Interestingly, studies have shown that early stomatal closure is a cause of lower tolerance in water-limited circumstances [54]. Within cluster 9, we observed 31 over-represented fusion GO terms, and, after applying a kappa score to define functional groups between GO terms, we identified 14 representative GO terms; these terms belong to 301 proteins (171 protein groups). Unlike the cyclic drought dataset, we only observed a few kinases (3 proteins; 3 protein groups) and transcription factors (2 proteins; 2 protein groups). Interestingly, one of these transcription factors (Potri.014G028200; up-regulated ~81 fold in B8) that is unique to this dataset is 58% similar in sequence identity to *A*. *thaliana ABF2*, which was recently shown to promote ABA-mediated chlorophyll degradation and leaf senescence by activating chlorophyll catabolic genes and senescence-associated genes [55]. This observation suggests that, on the one hand, protein abundances associated with chloroplast development are up-regulated under severe water deficit conditions after cyclic drought treatment, and, on the other hand, prolonged acute drought exhibits a gene regulation program promoting cell death and chloroplast degradation under severe water deficit conditions. Equally important, most associated proteins correlating with stomatal regulation in the acute drought treatment depict an obvious symptom of severe oxidative stress, resulting in the up-regulation of osmoprotective pathways [56] (e.g., Potri.T111300, *LEA4-5*; up-regulated ~335 fold), as well as the detoxification of reactive oxygen species [57] (e.g., Potri.008G149200, *ChlADR*; up-regulated ~464 fold) from an increase in lipid peroxidation of membranes. The rapid accumulation of reactive oxygen species under severe drought, consequently leading to lipid peroxidation, shows that the detrimental effect of water deficit is greater in the prolonged acute stress, suggesting that the cyclic drought plants are behaviorally more drought-tolerant.

### Identification of functional intersections and divergences between protein abundance behaviors and across experiments

**Fig 6:**
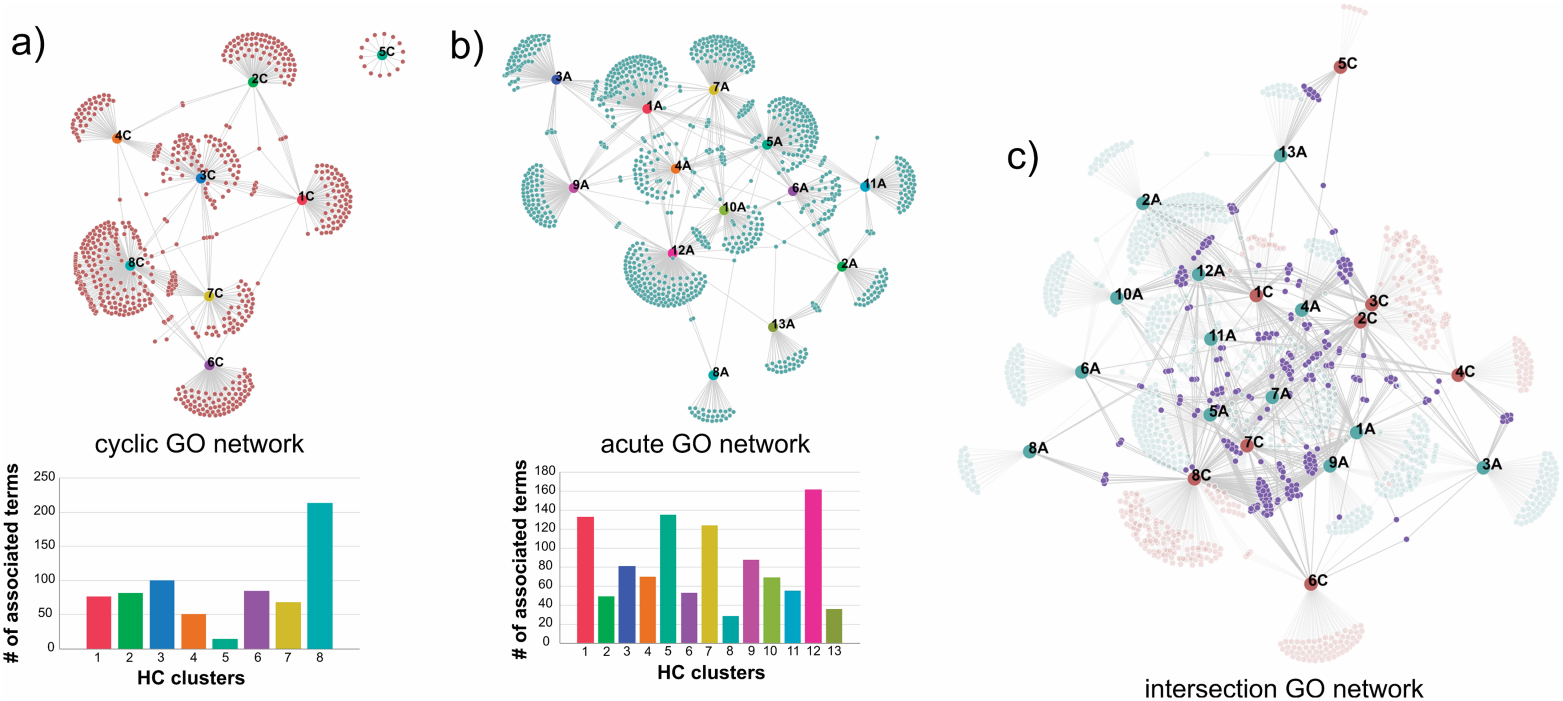
Multi-dimensional network of the protein abundance patterns to visualize functional intersections within and between drought experiments. For each network (a-c), the nodes represent either an identified hierarchical cluster number or a significantly enriched GO term. Overall, the node connectivity of significantly enriched GO terms is represented for each cluster as well as between clusters, which is conceptually like a Venn diagram. The **(A)** cyclic GO network and **(B)** acute GO network highlights the number of identified enriched GO terms for each cluster as well as the functional intersection, i.e., GO terms shared between each hierarchical cluster (HC). **(C)** To compare across the two water deficit treatments, the cyclic and GO networks were merged together to identify their functional intersections (purple nodes). Overall, there were 6 inter-experiment HC cluster pairs that shared 10 or more GO terms.

How plants perceive and respond to alterations in water status is modulated by the intensity, duration, and rate of progression of imposed drought. From the methodological point of view, this complexity poses several challenges to the compilation and integration of the available data. Moreover, the interpretation of large-scale proteomic datasets is not a trivial task. Therefore, to better interpret the dynamic cellular proteomes of these two different experiments, we developed a bioinformatics framework to assign the over-represented biological functional terms to the major protein abundance patterns identified in each experiment. This was achieved by using the major differential abundance patterns and identifying the significantly enriched gene ontology (GO) terms associated to those protein abundance responses. More specifically, using the hierarchical clusters from the cyclic drought (Fig 3) and acute drought (Figs 4 and 5) as target nodes and gene ontology terms as a source node, we created a network for both experiments; cyclic drought (Fig 6a) and acute drought (Fig 6b). This yielded a network topology from which we could deduce functional intersections (i.e., edges) between identified patterns of differential protein abundances occurring within and between the two drought experiments. Most of the biological processes were in distinctly separate functional modules and there were few highly connected GO terms between clusters. In general, GO terms were only highly interconnected between clusters when they shared similar differential abundance patterns, for example, cluster 7 and 8 share a high number of GO terms in the cyclic GO network. For information on each GO network, we illustrated the composition of the GO terms using ReviGO [58] (Figure A in S2 File).

By merging the cyclic and acute GO networks together (Fig 6c), we identified functional behavioral responses to drought that overlap both experimental datasets. Using this multi-dimensional network, we observed a substantial overlap in GO terms between cluster 8 and cluster 9 of the cyclic and acute drought experiments, respectively. As discussed above, the protein abundances associated within these clusters represent those that are up-regulated in response to severe drought in both experiments. As such, these GO represent a subset that are not only observed in both experiments, but also belong to proteins having similar differential responses. This bioinformatic framework has, therefore, produced a list of GO terms that now provide key insights into shared functional responses between two different drought experiments in *P*. *deltoides*. These shared biological processes and coping mechanisms are highlighted in Fig 2 in S2 File. Many of the over-represented GO terms were related to the regulation of stomatal closure and raffinose oligosaccharides, which are known to serve as a desiccation protectant [59]. Interestingly, we also identified GO terms belonging to proteins that have opposing behavioral responses between the two drought experiments (Figure B in S2 File), in which the associated protein abundances were up-regulated in the cyclic dataset and down-regulated in prolonged acute drought dataset. Most of the GO terms in this subset were associated with methylglyoxal, which plays an important role in promoting adaptation of plants growing under adverse environmental conditions [60]. Several of the proteins have been implicated in priming mechanism to have enhanced methylglyoxal defense system and their responses to drought were dependent on the duration of drought [61]. This observation provides additional insights into methylglyoxal as a potential biomarker for drought tolerance in plants, and demonstrates an importance in cyclic drought adaptation in *Populus*.

## Concluding remarks

In response to limited water supply, plants exhibit a highly variable response that is fine-tuned according to the duration and intensity of the associated stress. In this study, we highlight the dynamic changes of proteins across two different water deficit treatments. We identified quantitative patterns of differential proteins associated with functions related to reactive oxygen species scavenging, drought-relevant gene expression regulation, stomatal regulation, cell wall modulation and carbohydrate and energy metabolism. Previous investigations have repeatedly shown that it can be difficult to provide a reproducible interpretation of the mechanisms involved in drought response [9]. Through the integration of data from two different water deficit datasets, our study highlights that all water deficit-related investigations identify similar functional changes, in general, but, more importantly, the underlying proteins expectedly differ based on the experimental conditions. We coalesced the data from two experimental designs, in which one set of plants were expected to have manifested adaptive responses and the other are set of plants were less prepared for the onset of drought, to identify contrasts in protein abundance behaviors to provide new insights into specific proteins and their associated functions that manifest into an adapted response mechanism. For example, we observed that plants experiencing cyclic water deficit treatment, but not prolonged water stress, significantly up-regulate the abundances of a SNF1-related kinase, which is activated to promote an energy-saving program that maintains energy homeostasis while also gatekeeping important developmental transitions for optimal growth. Interestingly, this adaptive response was also observed at the transcript level in *Populus* “drought escape” and “drought avoidance” genotypes [12]. As described by Yildirim et al., both strategies are used to maintain high leaf water content by reducing plant leaf area.

While much is known about the effect of water deficit stress on plant growth and yield, the effects of limited water supply on the molecular level is less understood. The slow place in unraveling the molecular mechanisms is a result of how diverse a plant’s response can be. As demonstrated in this study, through large-scale and more integrative experiments, researchers can better identify the constituents that confer tolerance, rather than simply describing drought response mechanisms. Therefore, future investigations should not only include several water deficit treatments in the experimental design, but also more tissue types, given that the drought response happens at a whole-plant level.

## Supporting information

S1 File Supplemental Tables. Relevant supporting data can be found here and contains Tables A-F.
(ZIP)

S2 File Supplemental Figures. Relevant supporting figures can be found here and contains Figure A and Figure B.
(DOCX)
