# Supplementary material for: Quantitative proteome profile of water deficit stress responses in eastern cottonwood (Populus deltoides) leaves
Source: PLoS One. 2018 Feb 15;13(2):e0190019. doi: 10.1371/journal.pone.0190019 (PMC5813909; doi:10.1371/journal.pone.0190019)
Supplement: S2 File — Relevant supporting figures can be found here and contains Figure A and Figure B. (DOCX) [file pone.0190019.s002.docx]

**

**

**Figure A. REVIGO summary and visualization to prioritize enriched GO terms.** For the cyclic, acute and intersection (union) GO networks in the main text Fig. 5a-c, **(a)** we complemented these with summarized distributions of enriched GO terms across the GO term categories. In addition, we used the REVIGO webserver to reduce the redundancy of the enriched GO terms and provided REVIGO scatterplots for each representative dataset (i.e., cyclic, acute or union datasets) and each GO category: **(b)** biological processes, **(c)** cellular compartment, and **(d)** molecular function. Each scatterplot matrix shows the enriched GO terms in a two-dimensional space, which is derived by applying multi-dimensional scaling to a matrix of the GO terms’ semantic similarities. The bubble color indicates the position of each enriched GO term across one dimension of the semantic space and the bubble size indicates the -Log10 of the Holm-Bonferroni corrected p-value (see legend). For each plot, we highlighted a subset of the terms that represent some of the more significantly enriched functions.


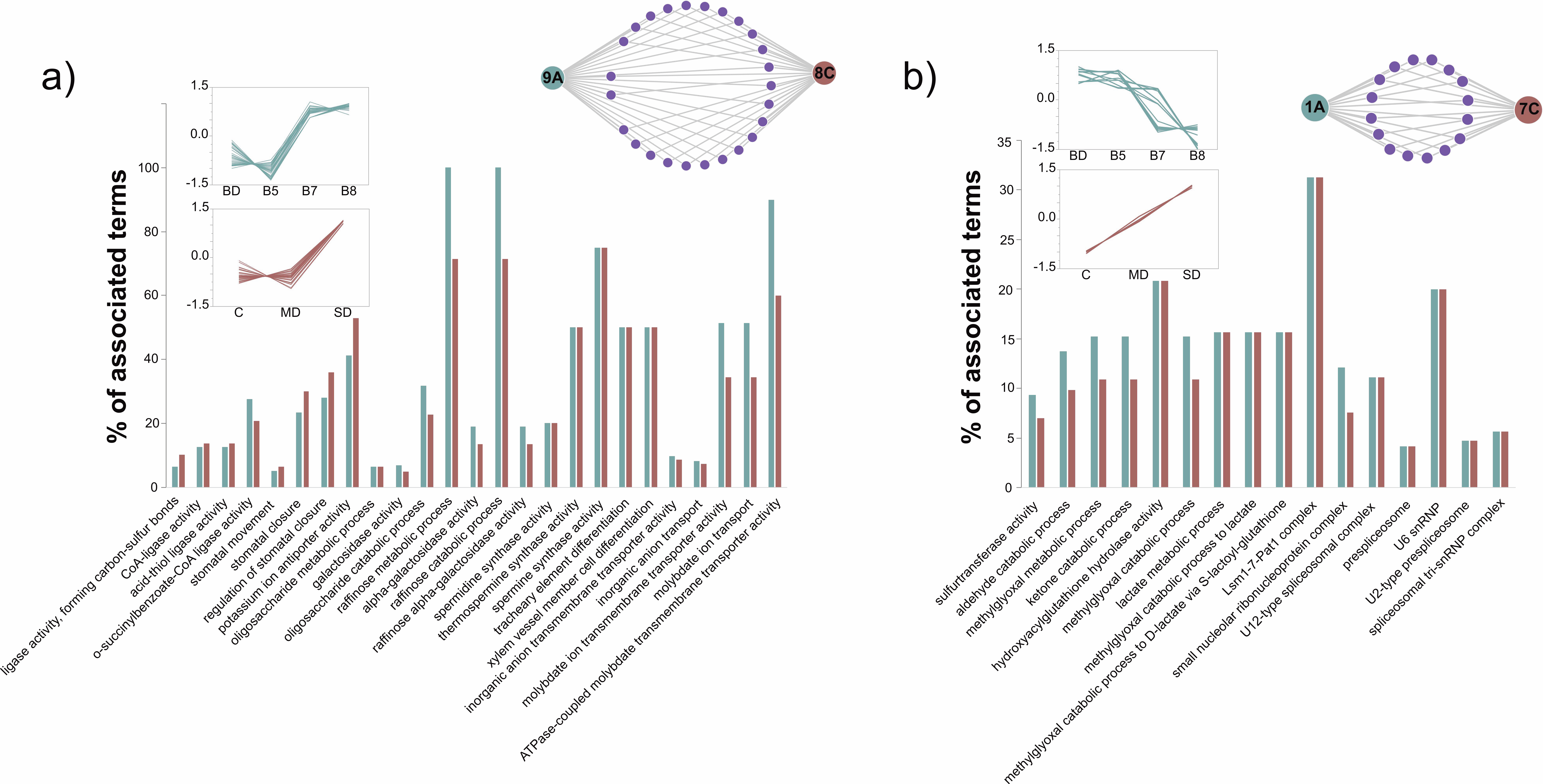


**Figure B. Functional intersections between cyclic and acute water deficit experiments.** We highlight two cluster associations in the intersection (union) GO network (Fig. 4c). **(a)** The inter-experiment HC cluster pair that had the most GO terms was between clusters 9 and 8 in the acute and cyclic network, respectively. **(b)** Another major functional overlap was between clusters 1 and 7 in the acute and cyclic network, respectively; however, the associated GO terms belong to proteins having an anti-correlative relationship in protein abundance trends from well-watered to severe water deficit. The percent of the total predicted associated terms in *Populus* is provided for each GO term. The abundance pattern for the proteins that are associated with these terms are illustrated as line plots, in which the y-axis is the standardized z-scores [(abundance - mean)/ standard deviation].
